# In vitro susceptibility profiles of invasive Candida bloodstream isolates to ten antifungal drugs in a southern area of China

**DOI:** 10.1099/jmm.0.002011

**Published:** 2025-05-12

**Authors:** Qian-Yu Sang, Yun-Hui Liao, Kai-Xuan Huang, Yin-Rong Xie, Yi-Hui Yao, Ping Chen, Xian-Ming Liang

**Affiliations:** 1 Department of Clinical Laboratory, Xiamen Hospital of Traditional Chinese Medicine, Beijing University of Chinese Medicine, Xiamen 361000, PR China; 2 Centre of Clinical Laboratory, Zhongshan Hospital of Xiamen University, School of Medicine, Xiamen University, Xiamen 361004, PR China; 3 Clinical Laboratory Branch, Xiamen Association of Integrative Chinese and Western Medicine, Xiamen 361000, PR China

**Keywords:** antifungal susceptibility, *Candida*, candidemia

## Abstract

**Introduction.** In recent years, with the increase of drug resistance of *Candida*, the incidence rate and mortality of candidemia have gradually increased, which has brought a huge economic and health burden to people.

**Gap Statement.** The epidemiological characteristics and antifungal drug sensitivity patterns in different regions have varied.

**Aim.** To analyse the distribution and antifungal susceptibility of *Candida* strains isolated from bloodstreams and provide a basis for the use of antifungal drugs for treatment.

**Methodology.** A total of 115 strains of *Candida* were collected from the bloodstream, and 28 strains of colonized *Candida albicans* were collected from the upper respiratory tract. *Candida* species were identified using matrix-assisted laser desorption/ionization time-of-flight technology. Antifungal susceptibility was assessed using broth microdilution combined with redox methods.

**Results.** There were eight types of *Candida* strains isolated from the bloodstream; *C. albicans* was the most common species (36.5%), followed by *Candida parapsilosis* (24.3%), *Candida glabrata* (17.4%) and *Candida tropicalis* (14.8%). There was no significant difference in the resistance of *C. albicans* to azole drugs between the bloodstream infection group and the upper respiratory tract colonization group, but there was a significant difference in the MIC values of micafungin and fluconazole, with *P* values of 0.017 and 0.003, respectively. Amphotericin B and echinocandins are the most susceptible drugs for all *Candida* species, but the MICs of echinocandins against *C. parapsilosis* are significantly higher than those of other *Candida* species. *Candida* (except for *C. glabrata*) is highly resistant to azoles, with *C. parapsilosis* showing resistance rates of 89.3% and 82.1% to itraconazole and posaconazole, respectively; the resistance rates of *C. tropicalis* are 100% and 94.1%, respectively.

**Conclusion.***C. albicans* remains the predominant pathogen responsible for candidemia. Although the resistance of *Candida* to antifungals is relatively stable, there are significant differences in the MICs of antifungal drugs against *Candida*, indicating the importance of strain identification in the treatment of candidemia. For empirical treatment, the use of echinocandin drugs is recommended.

## Introduction

With the widespread use of antibiotics, immunosuppressants and glucocorticoids in recent years, the incidence of candidemia has gradually increased [1], and the crude 30-day mortality rate is as high as 40% [2], causing heavy economic and disease burdens to patients. The most common pathogen causing candidemia is *Candida albicans*. In 2022, the WHO classified fungal pathogens into three priority groups based on the severity of their threat to human health, with *C. albicans* being the critical-priority group [3]. However, according to statistics, the incidence rates of non-*C. albicans* causing candidemia are increasing annually and are mainly due to *Candida tropicalis*, *Candida parapsilosis* and *Candida glabrata* [4,5]; at the same time, there are regional differences in the epidemiology of *Candida* species that cause candidemia [6,7]. However, laboratory-based techniques such as blood culture are still inefficient for the sensitive and rapid diagnosis of candidemia. Therefore, monitoring the trend of local epidemiology and understanding the sensitivity patterns of antifungal drugs are necessary to select appropriate antifungal drugs to improve patient prognosis. Furthermore, owing to the improper use of antifungal drugs and the lack of appropriate strategies to control their use, the resistance rate of *Candida* to fluconazole and echinocandins is slowly but steadily increasing [8]. This undoubtedly increases the difficulty of diagnosing and treating candidemia.

The clinical practice guidelines for managing candidiasis in the USA strongly recommend the use of echinocandins and azoles to treat candidemia [9]. In China, the resistance rate of *C. tropicalis* to fluconazole and voriconazole significantly increased from <8% in 2009–2010 to over 22% in 2013–2014 [10]. Fluconazole susceptibilities against *C. parapsilosis*, *C. albicans* and *C. glabrata* were 93.25%, 91.6% and 79.4%, respectively [6]. Recently, in clinical and laboratory studies, *Candida* with prior echinocandin exposure probably obtain resistance to echinocandins, particularly *C. glabrata*, and most studies suggest that this resistance is related to mutations in the catalytic subunit of glucan synthase (FKS) [8,11]. Amphotericin B and anidulafungin are the most susceptible drugs for all *Candida* species [6]. In many fungal infections, amphotericin B is the drug of choice, but it has a wide spectrum of adverse effects [12,13]. Although there are differences in drug sensitivity among various *Candida* species, the same species have a predictable pattern of antifungal sensitivity. Thus, it is particularly important to analyse local candidemia isolates to fully predict susceptibility patterns at the species level in specific hospitals or geographic regions. Currently, there is limited research on the drug resistance of *Candida* in bloodstream infections. The purpose of this study was to describe the species distribution and antifungal drug sensitivity of candidemia in our population and provide a basis for clinical experience in antifungal drug use.

## Methods

### Study population and protocol

This study collected clinical data from patients with *Candida* bloodstream infections and corresponding *Candida* isolates obtained from blood cultures at Zhongshan Hospital affiliated with Xiamen University from April 2010 to December 2023, which is a tertiary-level hospital that integrates medical treatment, teaching, prevention and healthcare. It admits more than 60,000 inpatients every year. Finally, 115 isolates from the bloodstream and 28 isolates from the upper respiratory tract were included in this study. The thawed strains were inoculated into Sabouraud medium (Zhengzhou Antu Biotechnology Co., Ltd.), placed in an incubator (Shanghai Xinmiao) at 35.0 °C for 18–24 h and then passaged twice. Species identification was performed using matrix-assisted laser desorption/ionization time-of-flight MS (Bruker Daltonik GmBH, Bremen, Germany). Briefly, single colonies were directly transferred to a target plate and overlaid with 1 µl of *α*-cyano-4-hydroxycinnamic acid matrix solution. The spectral acquisition was performed in linear positive ion mode with a mass range of 2,000–20,000 Da, using the manufacturer’s recommended settings. Instrument calibration was verified daily using Bruker Bacterial Test Standard. Mass spectra were analysed using MALDI Biotyper 4.1 software with a commercial database provided by Bruker. Identification scores ≥2.0 were considered reliable species-level identification. Antimicrobial susceptibility tests were performed using the colorimetric/turbidimetric method (Zhengzhou Antu Biotechnology Co., Ltd.).

This study was approved by the Institutional Ethics Committee of Xiamen Hospital of Traditional Chinese Medicine and was in compliance with national legislation and the Declaration of Helsinki guidelines.

### Antifungal susceptibility testing

The *in vitro* antifungal susceptibility tests of ten antifungal agents (anidulafungin, caspofungin, micafungin, amphotericin B, nystatin, itraconazole, fluconazole, voriconazole, posaconazole and 5-fluorocytosine) against *Candida* species were performed according to the manufacturer’s instructions. To prepare the inoculum, select isolated single colonies and prepare a standardized bacterial suspension equivalent to 0.5 McFarland units (0.5 MU) using sterile physiological saline, calibrated via a densitometer. Aspirate 10 µl of the prepared inoculum suspension, transfer it into a broth vial containing nutrient medium (16 ml) and homogenize by adding one drop (≈50 µl) of redox indicator. Finally, place the broth medium into the instrument. The instrument automatically completes the loading steps: using a robotic arm to quantitatively dispense 100 µl of inoculated broth into each well of the MIC panel, followed by incubation (35±2 °C, 18–24 h) and automated result reporting. *C. parapsilosis* (ATCC 22019) and *Candida krusei* (ATCC 6258) were included in each test as control isolates to monitor the accuracy and reproducibility of the susceptibility test procedure during each batch of experiments performed. Repeatability can be defined as the consistency of MIC values measured for at least nine out of ten drugs within the quality control range of ≥90%.

Result determination: (1) Amphotericin B and nystatin: compared with the positive control well, the antifungal drug coating well did not change from blue to red or purple, and the corresponding minimum coating concentration was the MIC value of the antifungal drug. (2) 5-Fluorocytosine, azole and echinocandins: the lowest coating concentration corresponding to the first micropore with slight colour intensity changes and significantly reduced precipitation (reduced by more than 50% compared with the positive control) is the MIC value. (3) A fully automatic microbial identification drug sensitivity analyser was used to automatically report MIC values and drug sensitivity results. The instrument employs a transmitted optical signal detection mechanism to monitor turbidity and/or chromogenic intensity changes in broth microdilution wells. Only MIC values are reported for drugs without MIC inflection points. According to the standards released by the CLSI, EUCAST and FDA in 2019, there is no MIC breakpoint for nystatin and 5-fluorocytosine, so only MIC values are reported. The standards for *Candida* susceptibility testing are shown in Table 1.

**Table 1.** Standard for *Candida* susceptibility tests

*Candida* species	Drug	S	SDD	I	R	ECV
*C. albicans*	Anidulafungin^*a*^	≤0.25	–	0.5	≥1	–
‍	Caspofungin^*a*^	≤0.25	–	0.5	≥1	–
‍	Micafungin^*a*^	≤0.25	–	0.5	≥1	–
‍	Amphotericin B^*b*^	≤1	–	–	>1	2
‍	Nystatin	–	–	–	–	–
‍	Itraconazole	≤0.064^*b*^	–	0.25–0.5^*c*^	>0.064^*b*^	–
‍	Fluconazole^a^	≤2	4	–	≥8	–
‍	Voriconazole^a^	≤0.12	–	0.25–0.5	≥1	–
‍	Posaconazole^b^	≤0.064	–	–	>0.064	0.06
‍	5-Fluorocytosine	–	–	–	–	–
*C. glabrata*	Anidulafungin^*a*^	≤0.12	–	0.25	≥0.5	–
‍	Caspofungin^*a*^	≤0.12	–	0.25	≥0.5	–
‍	Micafungin^*a*^	≤0.06	–	0.12	≥0.25	–
‍	Amphotericin B^*b*^	≤1	–	–	>1	2
‍	Nystatin	–	–	–	–	–
‍	Itraconazole	–	–	–	–	4
	Fluconazole	≤0.002^*b*^	≤32^*a*^	≤32^*c*^	≥64^*a*^	–
	Voriconazole	–	–	–	–	0.25
	Posaconazole	–	–	–	–	1
	5-Fluorocytosine	–	–	–	–	–
*C. tropicalis*	Anidulafungin^*a*^	≤0.25	–	0.5	≥1	–
	Caspofungin^*a*^	≤0.25	–	0.5	≥1	–
	Micafungin^*a*^	≤0.25	–	0.5	≥1	–
	Amphotericin B^*b*^	≤1	–	–	>1	2
	Nystatin	–	–	–	–	–
	Itraconazole^b^	≤0.125	–	–	>0.125	0.5
	Fluconazole^a^	≤2	4	4^*c*^	≥8	–
	Voriconazole^a^	≤0.12	–	0.25–0.5	≥1	–
	Posaconazole^b^	≤0.064	–	–	>0.064	0.12
	5-Fluorocytosine	–	–	–	–	–
*C. parapsilosis*	Anidulafungin^*a*^	≤2	–	4	≥8	–
	Caspofungin^*a*^	≤2	–	4	≥8	–
	Micafungin^*a*^	≤2	–	4	≥8	–
	Amphotericin B^*b*^	≤1	–	–	>1	1
	Nystatin	–	–	–	–	–
	Itraconazole^b^	≤0.125	–	–	>0.125	0.5
	Fluconazole^a^	≤2	4	4^c^	≥8	–
	Voriconazole^a^	≤0.12	–	0.25–0.5	≥1	–
	Posaconazole^b^	≤0.064	–	–	>0.064	0.25
	5-Fluorocytosine	–	–	–	–	–

*a*, *b* and *c* use the CLSI, EUCAST and FDA cut-off points, respectively, to determine drug sensitivity.

ECV, Epidemiological threshold; I, Intermediate; R, Resistant; S, Susceptible.

### Data statistics

The statistical analysis was carried out using IBM SPSS statistics version 29 (SPSS, Inc., Chicago, IL, USA) and GraphPad Prism version 10.0 (GraphPad Software, San Diego, CA, USA). MIC values that did not follow a normal distribution are reported as medians with ranges and 90th percentiles. The Mann–Whitney U test was used to statistically analyse the MIC values. Differences in *in vitro* antifungal susceptibility were determined using the chi-square test or Fisher’s exact test. *P*<0.05 was considered statistically significant.

## Results

### Information on strains

A total of 115 patients with candidemia came from 25 clinical departments. Among these patients, 34.8% were from the Intensive Care Unit (ICU), followed by hepatobiliary surgery (7.0%), urology surgery (6.1%) and the emergency department (5.2%), as well as the nephrology department (4.3%) and general surgery (4.3%) (Table 2). Eight different types of *Candida* were isolated from 115 strains, of which 42 (36.5%) strains were *C. albicans*, accounting for the highest proportion; *C. parapsilosis*, *C. glabrata* and *C. tropicalis* accounted for 24.3%, 17.4% and 14.8% of the isolates, respectively (Table 2).

**Table 2.** Patient clinical characteristics and information on the strains

Information	Age group (*n*)				Mean±sd	Total (%)
	≤**40**	41–**60**	61–**80**	＞80		
**Gender**						
Male	4	21	32	22	68.08±17.00	79 (68.7)
Female	4	15	12	5	61.39±19.31	36 (31.3)
**Departments**						
ICU		12	15	13		40 (34.8)
Hepatobiliary surgery		3	5			8 (7.0)
Urology		4	2	1		7 (6.1)
Emergency department			4	2		6 (5.2)
Nephrology department		3	2			5 (4.3)
General surgery	1	4				5 (4.3)
Emergency department-ICU	1		1	2		4 (3.5)
Cade health care department				4		4 (3.5)
Blood specialty	1	1	2			4 (3.5)
16 other clinical departments	5	9	13	5		32 (27.8)
***Candida* species**						
*C. albicans*	5	13	16	8		42 (36.5)
*C. parapsilosis*	1	11	8	8		28 (24.3)
*C. glabrata*	2	6	8	4		20 (17.4)
*C. tropicalis*		2	8	7		17 (14.8)
*Candida lusitaniae*		2	2			4 (3.5)
*Candida guilliermondii*		2				2 (1.7)
*Candida dubliniensis*			1			1 (0.9)
*Candida haemulonii*			1			1 (0.9)
**Total**	8	36	44	27		115 (100)

### Antifungal susceptibility of *C. albicans*

**Fig. 1.**
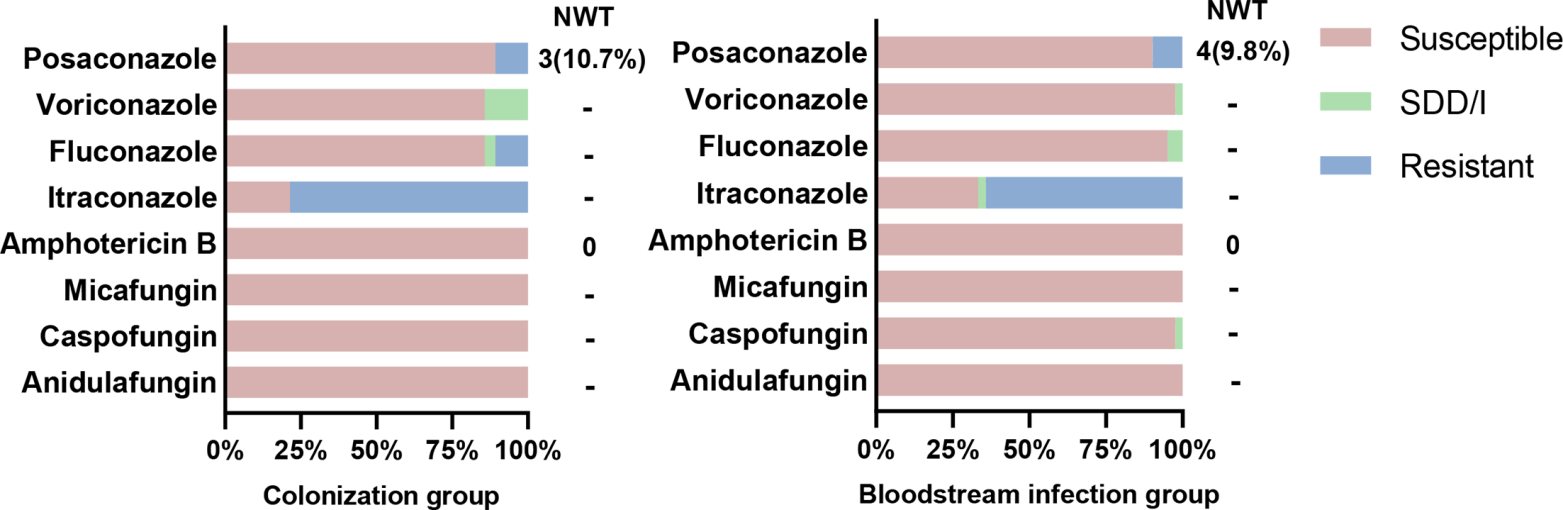
Antifungal susceptibility of *C. albicans*. Note: Bloodstream infection group (*n*=42) and colonization group (*n*=28). SDD/I, susceptible-dose-dependent; I, intermediate; -, no judgement inflection point; NWT, non-WT. Values adjacent to ‘NWT’ indicate the number and percentage of non-WT strains within resistant isolates.

To investigate whether there is a negative correlation between *Candida* virulence and antifungal resistance, we compared the drug sensitivity of *C. albicans* between the colonization group and the bloodstream infection group *in vitro* conditions (Fig. 1). *C. albicans* in the colonization group and *C. albicans* in the bloodstream infection group were both sensitive to echinocandins and amphotericin B, and all isolates were WT to amphotericin B. *C. albicans* have a certain degree of resistance to azole drugs, especially itraconazole, with 27 isolates (64.3%) in the bloodstream infection group and 22 isolates (78.6%) in the colonization group. However, there was no significant difference in drug sensitivity between the two groups.

**Fig. 2.**
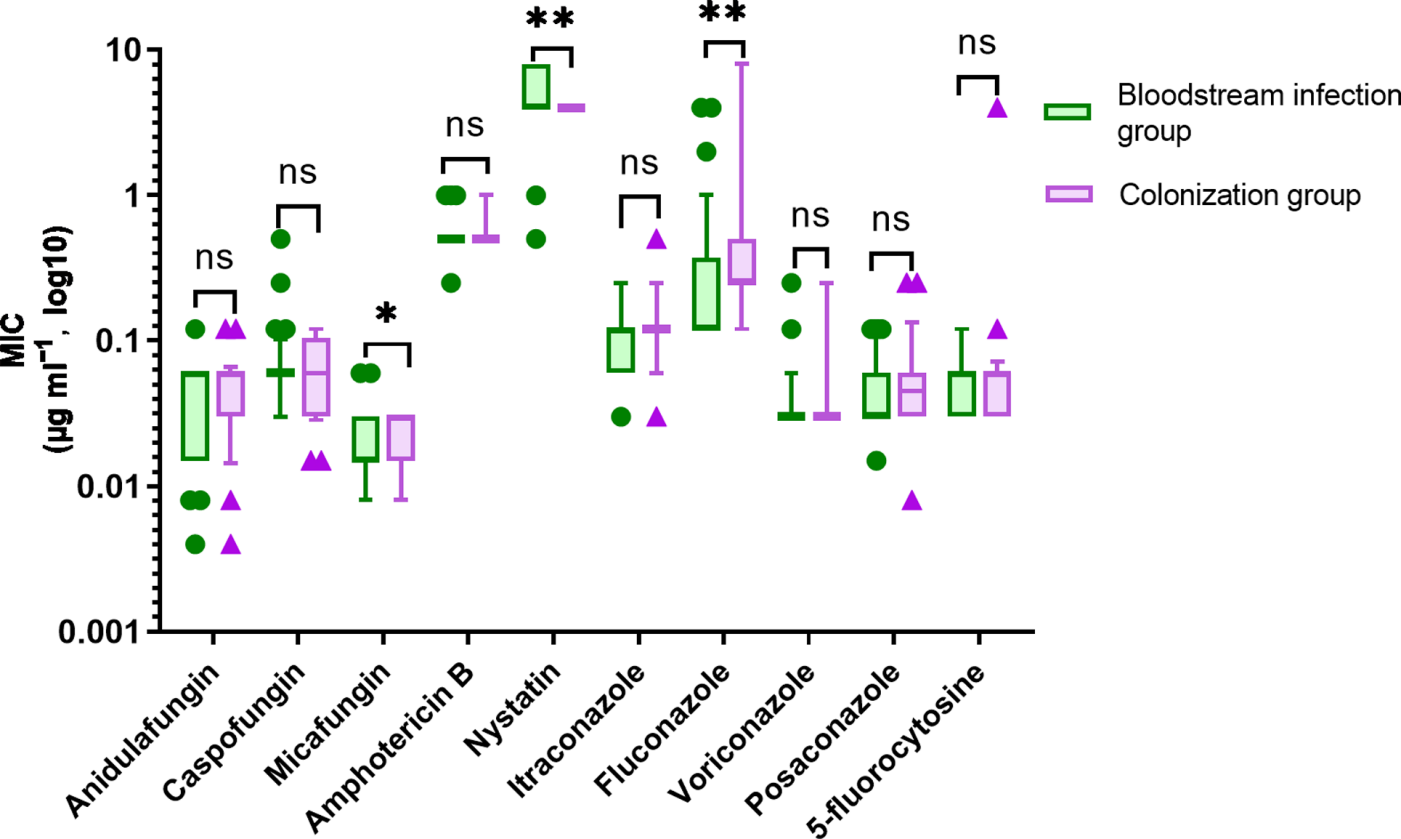
Distribution of MIC values for different antifungal agents against *C. albicans*. Note: Box-and-whisker plots depict the distribution of MICs for ten antifungal agents. The horizontal line within each box indicates the median, the box spans the 25th–75th percentiles, whiskers extend to 1.5× interquartile range and dots denote outliers. *P<0.05; **P<0.01; ns, not significant.

There was a statistically significant difference in the distributions of the MICs of micafungin, nystatin and fluconazole against *C. albicans* in the bloodstream infection group and colonization group, with *P* values of 0.017, 0.001 and 0.003, respectively (Table 3 and Fig. 2). The MIC_90_ of fluconazole against *C. albicans* in the bloodstream infection group was 8-fold greater than its MIC_50_, whereas the MIC_90_ of fluconazole against *C. albicans* in the colonization group was 32-fold greater than its MIC_50_, exhibiting the widest MIC range (0.12–8 µg ml^−1^). Nystatin had the narrowest MIC range (4 µg ml^−1^) against *C. albicans* in the colonization group, but the MIC range against *C. albicans* in the bloodstream infection group was 0.5–8 µg ml^−1^ (Table 3 and Fig. 2).

**Table 3.** Differences in MIC values of *C. albicans*

Drug	Bloodstream infection group			Colonization group			*P* value
	MIC range	MIC_50_	MIC_90_	MIC range	MIC_50_	MIC_90_	
Anidulafungin	≤0.004–0.12	0.06	0.06	≤0.004–0.12	0.06	0.06	0.600
Caspofungin	0.03–0.5	0.06	0.06	0.015–0.12	0.06	0.12	0.500
Micafungin	≤0.008–0.06	0.015	0.03	≤0.008–0.03	0.03	0.03	0.017
Amphotericin B	0.25–1	0.5	0.5	0.5–1	0.5	1	0.440
Nystatin	0.5–8	4	8	4	4	4	0.001
Itraconazole	0.03–0.25	0.12	0.25	0.03–0.5	0.12	0.25	0.447
Fluconazole	≤0.12–4	≤0.12	1	≤0.12–8	0.25	8	0.003
Voriconazole	≤0.03–0.25	≤0.03	0.06	≤0.03–0.25	≤0.03	0.25	0.650
Posaconazole	0.015–0.12	0.03	0.06	0.008–0.25	0.045	0.12	0.346
5-Fluorocytosine	≤0.03–0.12	0.06	0.12	≤0.03–4	0.06	0.06	0.422

*P* value: rank sum test *P* value; the unit of MIC is μg ml−1. MIC_50_, MIC required to inhibit 50% of isolates; MIC_90_, MIC required to inhibit 90% of isolates.

### Antifungal susceptibility of the first three types of non-*C. albicans*

**Fig. 3.**
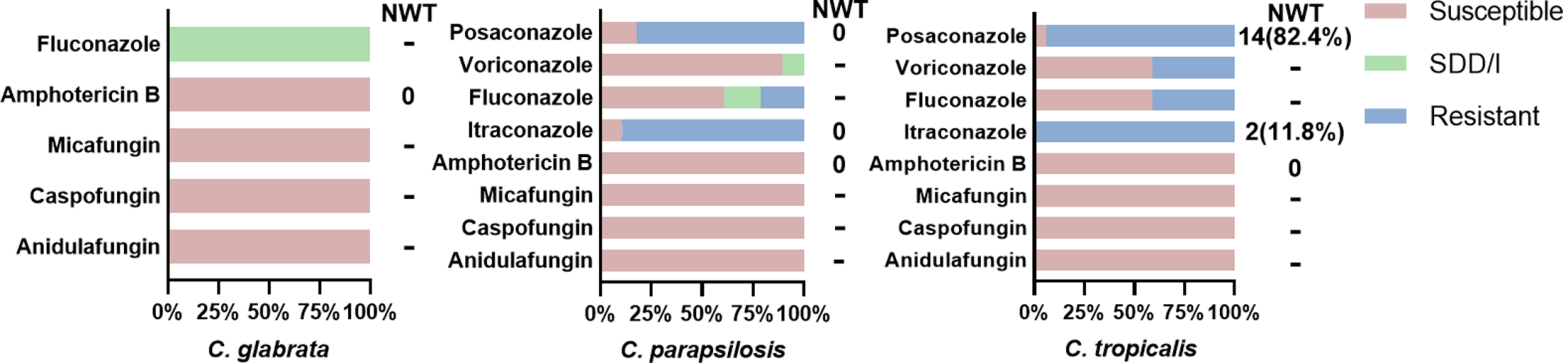
Antifungal susceptibility of three types of non-*C. albicans*. Note: This figure presents the antifungal susceptibility profiles of *C. glabrata*, *C. parapsilosis* and *C. tropicalis* isolates. Data normalized to 100% per species (total isolates: *C. glabrata*, *n*=20; *C. parapsilosis*, *n*=28; *C. tropicalis*, *n*=17). Values adjacent to ‘NWT’ indicate the number and percentage of NWT strains within resistant isolates. -, No judgement inflection point.

In this study, *C. glabrata*, *C. parapsilosis* and *C. tropicalis* were all susceptible to echinocandins and amphotericin B, with all isolates being WT to amphotericin B. Twenty (100%) *C. glabrata* isolates exhibited dose-dependent susceptibility to fluconazole. The resistance rates of *C. parapsilosis* to itraconazole, fluconazole and posaconazole were 89.3%, 21.4% and 82.1%, respectively, and 28 (100%) isolates were WT to itraconazole and posaconazole. The resistance of *C. tropicalis* to itraconazole, fluconazole, voriconazole and posaconazole was 100%, 41.2%, 41.2% and 94.1%, respectively, and 82.4% of the strains were non-WT (NWT) to posaconazole. No institutional breakpoints have been established for determining the *in vitro* susceptibility of these three non-*C. albicans* strains to 5-fluorocytosine (Fig. 3).

**Fig. 4.**
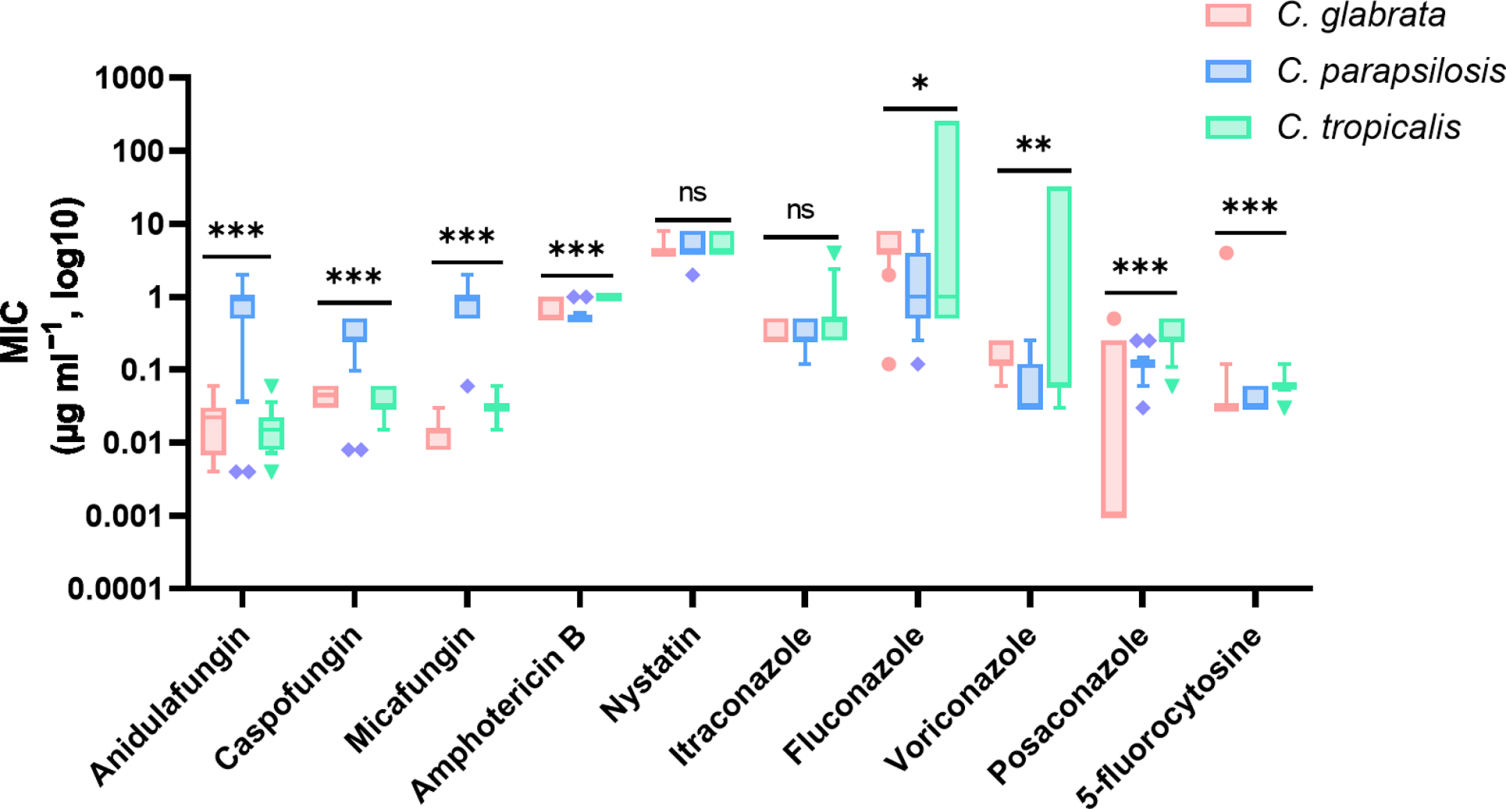
Distribution of MIC values for different antifungal agents against non*-C. albicans*. Note: Box-and-whisker plots depict the distribution of MICs for ten antifungal agents against non-*C. albicans*. The horizontal line within each box indicates the median, the box spans the 25th–75th percentiles, whiskers extend to 1.5× interquartile range and dots denote outliers. *P<0.05; **P<0.01; ***P<0.001; ns, not significant.

These three non-*Candida* species presented statistically significant differences in the distributions of MIC values for the other eight antifungal drugs except for nystatin and itraconazole (Table 4 and Fig. 4). Among them, *C. tropicalis* had the narrowest MIC range for amphotericin B (1 µg ml^−1^) and the widest MIC range for fluconazole (0.5–>256 µg ml^−1^); the MIC_90_ of fluconazole was 256-fold greater than its MIC_50_; and the MIC_90_ of voriconazole against *C. tropicalis* was 533-fold greater than its MIC_50_. The MIC_90_ of posaconazole (0.25 µg ml^−1^) against *C. glabrata* was 250-fold greater than its MIC_50_ (≤0.001 µg ml^−1^). In addition, the MIC_50_ of echinocandin against *C. parapsilosis* is almost ten times greater than that against *C. tropicalis* and *C. glabrata*.

**Table 4.** Differences in the MIC values of non*-C. albicans*

Drug	*C. glabrata*			*C. parapsilosis*			*C. tropicalis*			*P*value
	MIC range	MIC_**50**_	MIC_**90**_	MIC range	MIC_**50**_	MIC_**90**_	MIC range	MIC_**50**_	MIC_**90**_	
Anidulafungin	≤0.004–0.06	0.015	0.06	≤0.004–2	1	2	≤0.004–0.06	0.015	0.03	<0.001
Caspofungin	0.03–0.06	0.03	0.06	≤0.008–0.5	0.25	0.5	0.015–0.06	0.03	0.06	<0.001
Micafungin	≤0.008–0.03	0.015	0.03	0.06–2	1	2	0.015–0.06	0.03	0.06	<0.001
Amphotericin B	0.5–1	0.5	1	0.5–1	0.5	0.5	1	1	1	<0.001
Nystatin	4–8	4	8	2–8	4	8	4–8	4	8	0.177
Itraconazole	0.25–0.5	0.25	0.5	0.12–0.5	0.25	0.5	0.25–4	0.5	2	0.081
Fluconazole	≤0.12–8	4	8	≤0.12–8	1	8	0.5->256	1	>256	0.037
Voriconazole	0.06–0.25	0.12	0.25	≤0.03–0.25	≤0.03	0.25	≤0.03–32	0.06	32	0.002
Posaconazole	≤0.001–0.5	≤0.001	0.25	0.03–0.25	0.12	0.12	0.06–0.5	0.25	0.5	<0.001
5-Fluorocytosine	≤0.03–4	≤0.03	0.12	≤0.03–0.06	≤0.03	0.06	≤0.03–0.12	0.06	0.12	<0.001

*P* value: rank sum test *P* value; the unit of MIC is μg ml−1. MIC_50_, MIC required to inhibit 50% of isolates; MIC_90_, MIC required to inhibit 90% of isolates.

## Discussion

Candidemia, a common hospital-acquired bloodstream infection, has received increasing attention in recent years due to its high mortality rate, high treatment costs and increasing number of infections [14]. Echinocandins, which include anidulafungin, caspofungin and micafungin, constitute a first-line treatment for invasive candidiasis. In this study, all isolates were sensitive to echinocandins. Notably, the MIC_50_ of echinocandins against *C. parapsilosis* is almost ten times greater than that of other *Candida* species because *C. parapsilosis* has natural polymorphisms in the hotspots of FKS [15]. Despite higher MIC values, the prognosis of patients treated with echinocandins for *C. parapsilosis* was not affected [16], but some regions have shown the emergence of *C. parapsilosis* and *C. glabrata* resistance to echinocandins [17,18]. Reports indicate that repeated exposure to echinocandin drugs is a risk factor for the development of resistance in *C. parapsilosis* [19]. In addition, in accordance with the 2016 IDSA invasive candidiasis guidelines, for patients who previously received treatment with echinocandins and those infected with *C. glabrata* or *C. parapsilosis*, sensitivity testing for echinocandins should be considered [9]. Overall, most *Candida* species are still sensitive to echinocandins, but the guidelines for the clinical application of echinocandins should be more strict and standardized to avoid the development of drug-resistant *Candida*.

Azole antifungal drugs are widely used in clinical practice due to their economic, relatively safe and effective properties. However, the resistance of *Candida* to azole drugs is also the most common phenomenon. In our research, *C. albicans*, *C. parapsilosis* and *C. tropicalis* were the least sensitive to itraconazole, with drug resistance rates of 64.3%, 89.3% and 100%, respectively. *C. tropicalis* had the most severe resistance to azole drugs. Research has shown that the resistance mechanisms of *Candida* to azole drugs are mainly related to missense mutations in ERG11, which encodes lanosterol 14*α*-demethylase, and the overexpression of efflux pumps encoded by the CDR1, CDR2 and MDR1 genes [20,21]. A study based on the China Hospital Invasive Fungal Surveillance Net (CHIF-NET) in 2015 revealed that ~85.7% of *C. glabrata* isolates were susceptible-dose-dependent (SDD) to fluconazole and that 14.3% were resistant [22], which is similar to our finding that 20 *C. glabrata* isolates were SDD to fluconazole. These results indicate that although *Candida* is highly resistant to azole drugs, most strains, such as *C. albicans,* which are still sensitive to fluconazole and voriconazole, have not developed large-scale resistance. Therefore, the clinical use of triazole drugs must be approached with care to minimize overuse.

Polyene drugs, represented by amphotericin B, are classic antifungal drugs. Compared with those resistant to echinocandins and triazole drugs, *Candida* strains resistant to amphotericin B are extremely rare [23]. In our study, all isolates of *Candida* were WT and susceptible to amphotericin B. Although amphotericin B has good therapeutic effects in clinical practice, its clinical application is not popular, mainly because of its low oral bioavailability and dose-dependent toxic effects on the host caused by the similarity between ergosterol and cholesterol [24]. 5-Fluorocytosine is an antifungal drug that targets nucleic acid biosynthesis and is commonly used in combination with amphotericin B to treat invasive candidiasis, but it has toxic side effects on liver and kidney function as well as the blood system [25]; therefore, its clinical use is limited.

As a component of the commensal microbiota, *C. albicans* usually colonizes the mucous membranes of normal individuals in the form of yeast [26]. The transition from yeast to the hyphal form is a transition into a pathogenic form, and hypha is one of the important factors in the virulence of *C. albicans* [27]. Moreover, the cell wall structure of *C. albicans* also changes, which is related to significant differences in adaptation and resistance to antifungal drugs [27,28]. However, there was no significant difference in the sensitivity of *C. albicans* to the ten antifungal drugs between the bloodstream infection group and the colonization group in this study, which may be related to the limited number of *C. albicans* isolates included.

Consistent with the findings of most studies, the incidence of candidemia usually happened in elderly people and ICU departments. This may be related to the use of broad-spectrum antibiotics, prolonged intensive care unit time, mechanical ventilation, total parenteral nutrition, the use of central venous catheter medical devices and other diseases leading to weakened immunity [29]. The top four bloodstream infections of *Candida* in our study were *C. albicans* (36.5%), *C. parapsilosis* (24.3%), *C. glabrata* (17.4%) and *C. tropicalis* (14.8%). This result is slightly different from the data released by the CHIF-NET: the detection rates of *C. albicans*, *C. parapsilosis*, *C. tropicalis* and *C. glabrata* in bloodstream infections from 2009 to 2014 accounted for 32.3%, 28.90%, 17.50% and 11.50%, respectively [30], which may be due to regional differences. In conclusion, similar to most studies [6,7], *C. albicans* is still the principal species responsible for candidemia.

There are several limitations to this study. First, it was a single-centre study with a relatively small sample size. Second, research on resistance-related genes and mechanisms is lacking. Third, this retrospective study did not include a large pool of controls at random. We look forward to multicentre prospective studies being planned to overcome those shortages in the future.

*C. albicans* remains the predominant pathogen responsible for candidemia. *Candida* species exhibit high resistance to azole antifungals (particularly itraconazole) but remain susceptible to echinocandins and amphotericin B. In summary, the *Candida* strains isolated from our population have relatively stable resistance to antifungal drugs. Therefore, in critical situations, appropriate empirical medication can be used to improve treatment efficiency, but blind empirical medication should be avoided to reduce the occurrence of drug-resistant strains.

## Abbreviations

NWT: non-WT
SDD: susceptible-dose-dependent
